# Telomere Length Modulation in Human Astroglial Brain Tumors

**DOI:** 10.1371/journal.pone.0064296

**Published:** 2013-05-14

**Authors:** Domenico La Torre, Alfredo Conti, M′Hammed Aguennouz, Maria Grazia De Pasquale, Sara Romeo, Filippo Flavio Angileri, Salvatore Cardali, Chiara Tomasello, Concetta Alafaci, Antonino Germanò

**Affiliations:** 1 Department of Neurosciences, University of Messina School of Medicine, Messina, Italy; 2 Department of Medical Oncology, University of Messina School of Medicine, Messina, Italy; Beijing Tiantan Hospital, Capital Medical University, China

## Abstract

**Background:**

Telomeres alteration during carcinogenesis and tumor progression has been described in several cancer types. Telomeres length is stabilized by telomerase (h-TERT) and controlled by several proteins that protect telomere integrity, such as the Telomere Repeat-binding Factor (TRF) 1 and 2 and the tankyrase-poli-ADP-ribose polymerase (TANKs-PARP) complex.

**Objective:**

To investigate telomere dysfunction in astroglial brain tumors we analyzed telomeres length, telomerase activity and the expression of a panel of genes controlling the length and structure of telomeres in tissue samples obtained *in vivo* from astroglial brain tumors with different grade of malignancy.

**Materials and Methods:**

Eight Low Grade Astrocytomas (LGA), 11 Anaplastic Astrocytomas (AA) and 11 Glioblastoma Multiforme (GBM) samples were analyzed. Three samples of normal brain tissue (NBT) were used as controls. Telomeres length was assessed through Southern Blotting. Telomerase activity was evaluated by a telomere repeat amplification protocol (TRAP) assay. The expression levels of TRF1, TRF2, h-TERT and TANKs-PARP complex were determined through Immunoblotting and RT-PCR.

**Results:**

LGA were featured by an up-regulation of TRF1 and 2 and by shorter telomeres. Conversely, AA and GBM were featured by a down-regulation of TRF1 and 2 and an up-regulation of both telomerase and TANKs-PARP complex.

**Conclusions:**

In human astroglial brain tumours, up-regulation of TRF1 and TRF2 occurs in the early stages of carcinogenesis determining telomeres shortening and genomic instability. In a later stage, up-regulation of PARP-TANKs and telomerase activation may occur together with an ADP-ribosylation of TRF1, causing a reduced ability to bind telomeric DNA, telomeres elongation and tumor malignant progression.

## Introduction

**T**elomeres consist of long tandem arrays of TTAGGG repeats, bound by proteins, placed at the end of linear chromosomes, which are involved in several essential biological functions.[1], [2] These non-coding telomeric repeats represent a buffer zone preventing the adjacent coding region of the genome from erosion. In normal human cells, telomeres decrease by some 5–20 repeats with every cell division.[3] Therefore telomere shortening limits the number of times a cell can divide.[4] Hence, they can regulate the onset of replicative senescence in the somatic cells.[5]–[7]

In human cells, several pathways regulating telomeres length have been identified. The most important is regulated by telomerase, that catalyzes extension of 5′-ends of the lagging DNA strand by adding TTAGGG repeats onto the telomeres using its intrinsic RNA as template for reverse transcription.[8] Two major subunits of the human telomerase core complex have been identified, namely h-TERC and h-TERT. The former serves as a template for telomeres elongation; instead, the latter subunit (h-TERT) contains a reverse transcriptase domain that catalyzes this reaction.[9]

The length and structure of telomeres are also controlled by a variety of proteins. Collectively, these telomeric proteins protect telomere integrity and function, connect DNA damage/repair network with the controls of cellular senescence, monitor telomere homeostasis and modify the access of telomerase to telomeres. The two major proteins are the duplex TTAGGG repeat-binding factors 1 and 2 (TRF1 and TRF 2) that are localized at telomeres.[10] These proteins play a key role in the maintenance of telomere function and structure modifying telomerase activity.[11]–[13] Recent evidence shows that TRF1 interacts with other telomere-binding molecules.[14] TRF1 accepts adenosine diphosphate (ADP)-ribosylation catalyzed by the tankyrase-poli-ADP-ribose polymerase (TANKs-PARP) complex. The ADP-ribosylation of TRF1 reduces its ability to bind telomeric DNA, allowing telomerase to elongate telomeres and extending the cellular life span.[15]–[17]

The alteration of telomere length homeostasis affects telomere structure and leads to genomic instability by generating chromosome end-to end fusion and chromosomal abnormalities.[18] It has been demonstrated that telomeres shortening could initiate successive events, such as aberrant fusion or recombination of the end of chromosomes, genomic instability, loss of cell growth control, and finally cancer development.[19], [20]

The phenomenon of telomeres alteration during carcinogenesis and cancer progression is well known and established at the molecular level.[21]–[23] Nevertheless, studies focused on the analysis of telomere dysfunction in astroglial brain tumors are missing. The present study was designed to investigate the expression levels of a panel of genes controlling the length and structure of telomeres in human astroglial brain tumors with different grade of malignancy (WHO Grade 2–4). We analyzed telomeres length, telomerase activity and the expression levels of TRF1, TRF2, h-TERT and TANKS-PARP complex in tumor samples obtained *in vivo*, investigating the presence of a specific genes expression profile during the different stages of tumor progression, from low grade astrocytomas to glioblastoma.

## Materials and Methods

### Ethics Statement

The manuscript has been submitted to the Ethics Committee on February 21, 2011. The above mentioned Committee issued a formal written waiver for the need of ethics approval. All patients signed a written informed consent for the purpose of publication of clinical data, according to the internal regulation. Results were analyzed anonymously.

### Patient population

Tumors samples, histologically verified as grade 2–4 astrocytomas, were obtained in adult patients who underwent craniotomy for microsurgical tumor resection. All tumors were located in the supratentorial compartment. Only patients who had undergone gross total resection (more than 95% of the tumor volume) were eligible for the study. Summary of demographic and clinical data are reported in *table 1*. Samples obtained from single or multiple stereotactic biopsies were not included in the present study. We carefully excluded tumors containing components that were suspicious of oligodendroglioma. No case of recurrent tumors and no patient who underwent radio and/or chemotherapy before surgery were employed in the present study.

**Table 1:** Summary of Demographic and Clinical Data of Patients.

Tumor types	WHO grade	Number of patients	Female	Male	Age (Yrs)	KPS	OS (weeks)
**LGA**	II	8	3	5	41.5 ± 6.5	95 ± 7.5	194.25 ± 38.89
**AA**	III	11	6	5	65.4 ± 8.6	89.09 ± 12.2	107.3 ± 30.3
**GBM**	IV	11	5	6	66.2 ± 6.1	88.18 ± 10.7	62.3 ± 14.8
**NBT**	/	3	1	2	43±3.2	/	/
**TOTAL**	**/**	30	14	16	59.3 ± 12.9	90.3 ± 10.6	114.03 ± 59.7

**All values are expressed as mean ± standard deviation.**

**Abbreviations used:** LGA, low grade astrocytoma; AA, anaplastic astrocytoma; GBM, glioblastoma multiforme; WHO: World Health Organization; M: male; F: female; KPS: Karnofsky Performance Scale; OS: overall survival; NBT: normal brain tissue.

### Tissue Samples

All tumor tissue samples were obtained from resection specimens, within 15 minutes from surgical tissue removal. Specimens were taken from viable areas of tumor, avoiding areas of gross necrosis. Three to seven anatomically separate areas of tumor tissue were sampled from each resection specimen, according to the volume of resected tissue available. Tumor samples were placed in cryovials and immediately flash-frozen in liquid nitrogen in the operating room and stored at −70°C. Tissue samples adjacent to the frozen tissue, as well as additional tissue submitted *in toto* from the resection specimens, were both used for histological typing and grading according to WHO criteria. Three samples of normal brain tissue were used as controls. Non-neoplastic brain tissue samples were derived from the temporal lobes of patients surgically treated for temporal lobe epilepsy, histologically verified as normal cortex and white matter.

### Telomere length analysis

Terminal restriction fragment (TRF) length measurements in tumor specimens and in normal samples were obtained by using the *Telo* TTAGGG telomere length assay kit (Roche Diagnostics, Milan, Italy), according to the manufacturer's recommendations. The intensity of the hybridization was evaluated by densitometric analysis with Quantity One software (Bio-Rad Laboratories, Hercules, CA) and mean TRF length of a sample was estimated according to the formula as described by Harley CB at al.[5]

### Telomerase activity Assay

Telomerase activity was measured by a telomere repeat amplification protocol (TRAP) assay using TeloTAGGG Telomerase PCR ELISA plus® (Roche, Mannheim, Germany) according to the manufacturer's recommendations. The relative telomerase activity (RTA) within different samples was calculated using the following formula: RTA = (*A*_sample_/*A*_sample,IS_)/(*A*_TS8_/*A*_TS8,IS_)×100%, where *A*_sample_  =  absorbance of sample; *A*_sample,IS_  =  absorbance of internal standard of sample; *A*_TS8_  =  absorbance of control template; and *A*_TS8,IS_  =  absorbance of internal standard of control template.

### Extraction of total RNA and Real-Time Quantitative PCR

Total RNA was extracted from each specimen using TRIizol reagent and purified with RNA Purification kit (Rneasy Mini Kit clumns- Qiagen). The quality and quantity were checked respectively on agarose gel and spectrophotomety. Three µg of total RNA from each sample was reverse-transcribed by Archive kit (Applied Biosystems Milan, Italy). Generated cDNA was used as template for real time quantitative PCR analysis using gene expression products according to the manufacturers recommendations (Applied Biosystems). All reactions were performed with a 7300 Sequence Detection System apparatus (Applied Biosystems) to measure and compare the mRNA level of TERF-1, TERF-2, TNKS, PARP1, h-TERT, and β-actin (as an endogenous control).

Relative quantification (RQ) for these genes was expressed as fold variation over control, and calculated by the ΔΔCt method, using normal brain tissue (control) as calibrators.

### Proteins extraction, electrophoresis (SDS-PAGE) and immunoblotting

Frozen tumor tissues (∼50 mg) was harvested by homogenization with a Potter homogenizer in a 15 volumes ice-cold triple detergent lysis buffer.

Immunoblots were probed with goat polyclonal antibody anti TRF1 and TANKS, mouse monoclonal antibody TRF2, PARP1 and β-actin (Santa Cruz Biotechnology Inc., Santa Cruz, California, USA). Following incubation with primary antibody (1∶200) at RT for 2 hours, blots were incubated with a secondary antibody: mouse- anti-goat and rabbit – anti-mouse IgG, (1∶1000; DAKO) conjugated to peroxidase at RT for 1 hour. Enhanced chemiluminescence reagents were used to visualize immunolabeling on Kodak Biomax ML chemiluminescent film. (ECL, Amersham biosciences, Little Chalfont, Buckingamshire, UK).

### Quantification of telomeric proteins

Semi-quantitative evaluation of protein levels detected by immunblotting was performed by computer-assisted densitometric scanning (AlphaImager 4.2 Digital Imaging System, Italy). Different time of exposure were used for each blot (15–25 seconds), and longer exposures were performed in an attempt to detect very low levels of proteins in normal brain tissue. Data were acquired as integrated densitometric values and expressed as percentages of the densitometric levels obtained on scans from normal brain tissue used as control visualized on the same blot (ADU - Arbitrary Densitometric Unit). We used two different negative controls for each blot.

### Statistical Data Analysis

Statistical analysis was accomplished using the unpaired Student t-test to compare the expression levels of mRNA of TRF1, TRF2, h-TERT, Tankyrase, PARP1 as quantified on real time RT-PCR and telomere length on southern blotting. The Spearman nonparametric correlation test was used to assess the correlation among mRNA expression levels and the nominal variables (WHO grade). Computer software programs (INSTAT [version 3.0] and PRISM [version 4.0]; GRAPHPAD, San Diego, CA and MedCalc [version 7.2.1.0]) were used to perform the data analysis. A probability value less than 0.05 was considered statistically significant. All values where expressed as mean ± standard deviation. All tests are two-tailed; values are expressed as mean ± standard deviation.

## Results

### Telomere length in normal brain and astroglial brain tumors

**Figure 1:**
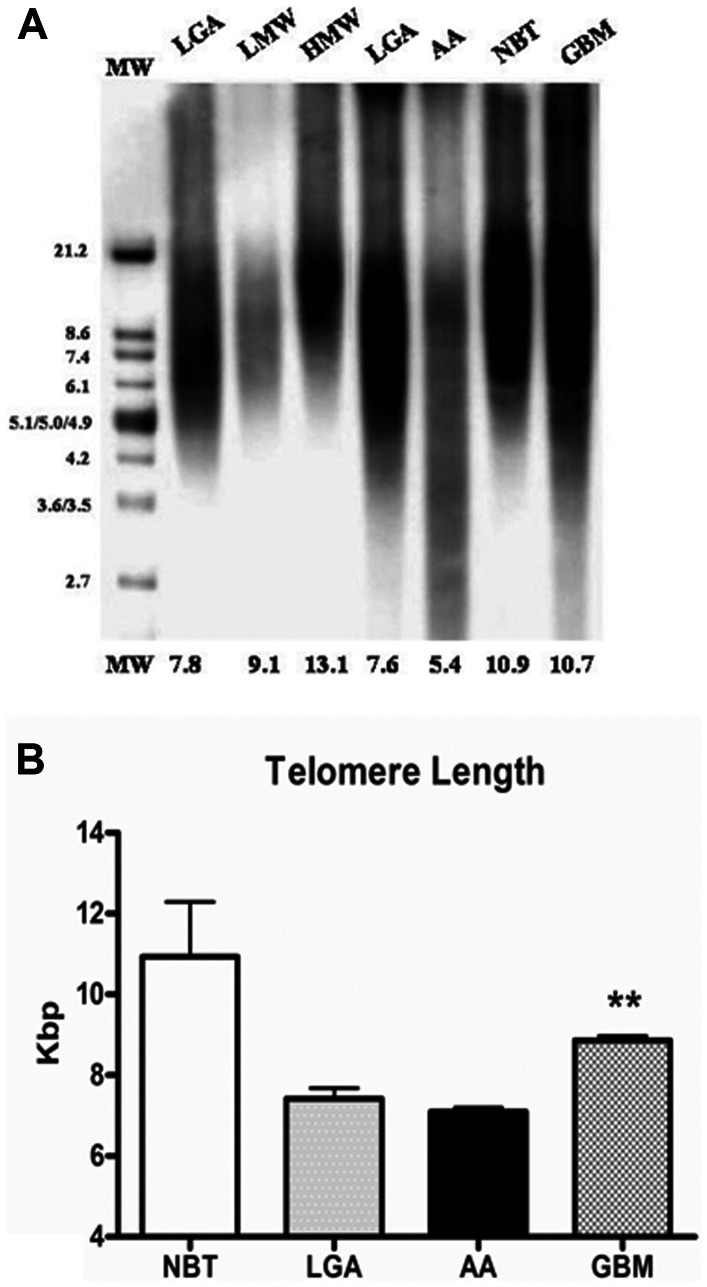
Terminal Restriction Fragment Analysis. Representative autoradiogram of southern blot analysis. Telomere length was reduced in astroglial tumors as compared with NBT, but elongated telomere was frequently found in GBMs as compared with both AAs and LGGs (p<.001). ***A*** Bar Graph. Shows telomere length (x axis) in different grade of astrocytomas and controls (y axis). Error bars indicate standard deviation. ** indicates significance of p<.001. **Abbreviations**: Kbs: kilobase pair; NBT: normal brain tumor; LGA: Low grade Astrocytoma; AA: Anaplastic Astrocytoma; GBM: Glioblastoma Multiforme; LMW: Low molecular weight; HMW: High molecular weight; MW: Molecular Weight.

Telomere length varied in astroglial brain tumors with different grade of malignancy. Telomere length was 7.4±0.23 Kbs in LGGs; 6.9±0.14 Kbs in AAs; 9.2±0.5 Kbs in GBMs; 10.93±2.35 Kbs in NBT (Figure 1). Elongated telomere was frequently found in GBMs as compared with both AAs and LGGs (p<.001). No statistical differences in telomere length between AAs and LGGs were observed. Interestingly, telomeres in tumor specimens were always shorter as compared with NBT.

### Telomerase activity and h-TERT expression

**Figure 2:**
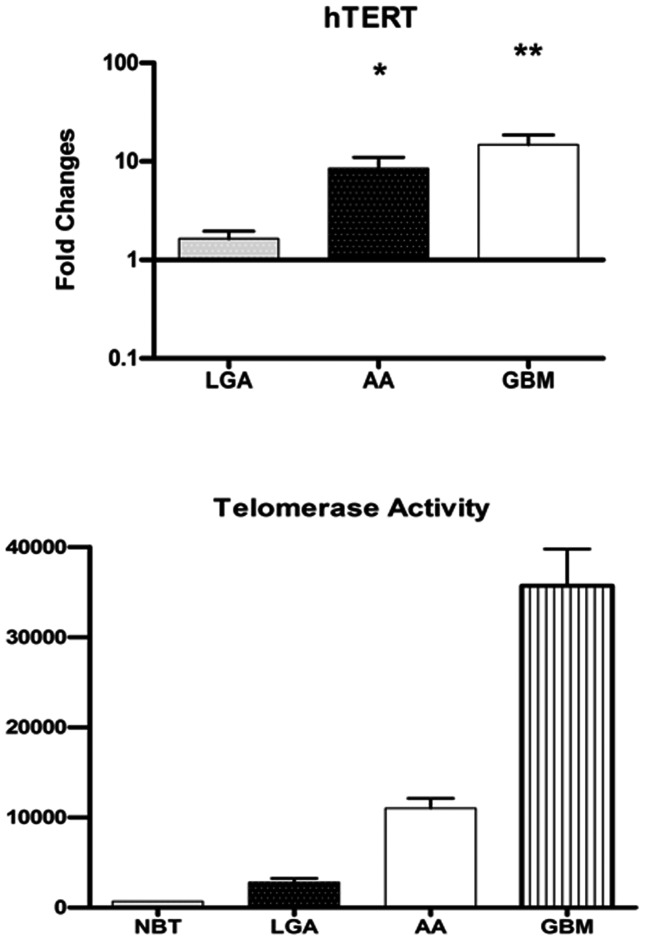
Telomerase activity and h-TERT mRNA expression. Telomerase activity significantly correlates with h-TERT mRNA expression and WHO in astroglial brain tumors. The h-TERT expression levels in GBMs resulted statistically higher as compared with those in LGAs. The expressions in AAs differed significantly as compared with that in LGAs. A correlation was found between h-TERT and WHO grading (p<.001). Telomerase activity is expressed as relative telomerase activity (RTA) according to the formula showed in the methods. Error bars indicate standard deviation. * p<.05; ** p<.001. **Abbreviations**: NBT: normal brain tumor; LGA: Low grade Astrocytoma; AA: Anaplastic Astrocytoma; GBM: Glioblastoma Multiforme; h-TERT human telomerase reverse transcriptase.

Telomerase activity (TA) in NBT was weak or undetectable using the TeloTAGGG Telomerase PCR ELISA plus. Telomerase activity was 3.9-fold in LGAs, 15.75-fold in AAs, and 51-fold in GBMs as compared to normal control values. Telomerase activity significantly correlates with h-TERT mRNA expression and WHO grade (Figure 2). h-TERT mRNA expression was 1.71±0.90 in LGAs, 8.45±8.49 in AAs, 14.8±12.46 in GBMs. The expression levels in GBMs resulted statistically higher as compared with those in LGAs (P = .009). The expressions in AAs differed significantly as compared with that in LGAs (P = .04). A correlation was found between h-TERT and WHO grading (P<.001). Conversely, no statistical differences in telomere length between h-TERT and Telomere length were observed. A correlation was also found between expressions of hTERT mRNA and PARP-1 mRNA (P = .008), and Tankyrase mRNA (P = .058).

### Telomere-associated proteins

#### Down-regulation of TRF1 and 2 and TANKS-PARP up-regulation occurs along with malignant progression in astroglial brain tumours

**Figure 3:**
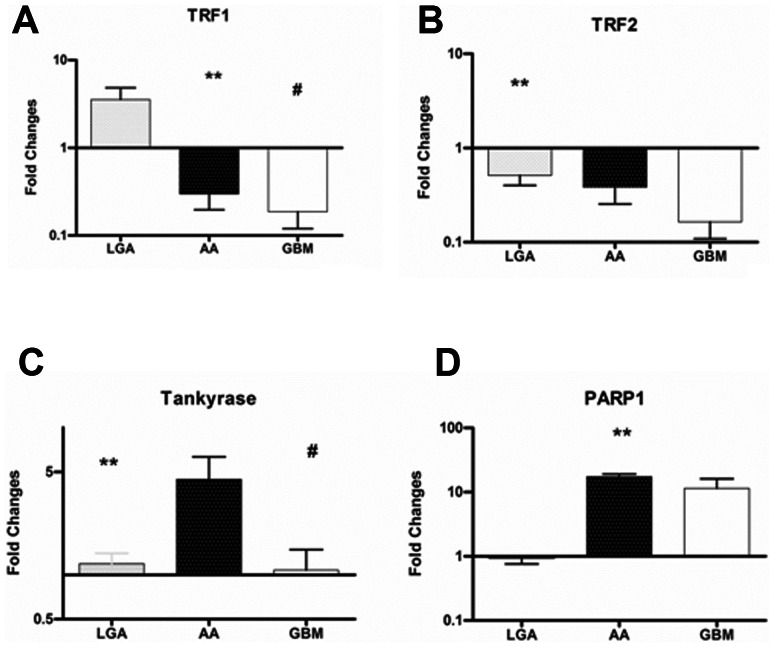
Expression levels of Telomere-associated proteins among tumors with different grade of malignancy. Quantitative mRNA expression levels of TRF1, TRF2, TANKs, and PARP-1 was determined by real-time RT-PCR analysis. Relative quantification for these genes was expressed as fold variation over control, and calculated by the ΔΔCt method, using control samples (actin) as calibrators. ***A*** TRF1 expression levels progressively decrease from LGAs to GBMs. The expression in LGAs was significantly higher than that in both GBMs (P = .007) and AAs (P = .009). ***B*** TRF2 expression levels in LGAs resulted statistically higher as compared with those in GBMs (P = .008), and showed a tendency toward a lower expression in AAs as compared with LGAs ***C*** Increased expression of TANKs resulted in AAs compared with LGAs and GBMs. ***D*** PARP1 mRNA expression levels resulted statistically higher in AAs as compared with those in LGAs. Moreover, PARP expression in GBMs showed a tendency toward the higher expression as compared with LGAs. **Abbreviations**: LGA: Low grade Astrocytoma; AA: Anaplastic Astrocytoma; GBM: Glioblastoma Multiforme; TRF1, Telomeric repeat-binding factors 1; TRF2, Telomeric repeat-binding factors 2; PARP1, Poly (ADP-ribose) polymerase 1.

Summary of biomolecular data are reported in table 2. TRF1 mRNA expression was 3.54±3.65 in LGAs, 0.30±0.34 in AAs; 0.19±0.22 in GBMs. The expression levels in LGA were statistically different as compared with those in GBMs (P = .007). The expression in LGAs was significantly higher than that in AAs (P = .009) (Figure 3 A). An inverse correlation was found between expressions of TRF1 and WHO grade (P = .006).

**Table 2:** Summary of biomolecular data.

	NBT	LGA	AA	GBM
Telomere length*	10.93±2.35	7.4 ± 0.23	6.9 ± 0.14	9.2 ± 0.5
**h-TERT**		1.71±0.90	8.45±8.49	14.8±12.46
**TRF1**		3.54 ± 3.65	0.30 ± 0.34	0.19 ± 0.22
**TRF2**		0.51±0.22	0.38 ±0.43	0.16 ±0.19
**Tankirasi**		1.28±0.58	4.46 ± 6.23	1.08±1.35
**PARP1**		0.97±0.59	17.5±5.69	11.5±15.5

**Abbreviations used:** LGA, low grade astrocytoma; AA, anaplastic astrocytoma; GBM, glioblastoma multiforme; * values in kilobase pairs. h-TERT human telomerase reverse transcriptase; TRF1, Telomeric repeat-binding factors 1; TRF2, Telomeric repeat-binding factors 2; TANKs, Tankyrase; PARP1, Poly (ADP-ribose) polymerase 1.

The TRF2 mRNA expression was 0.51±0.22 in LGAs, 0.38±0.43 in AAs; 0.16±0.19 in GBMs. The expression levels in LGAs resulted statistically higher as compared with those in GBMs (P = .008) (Figure 3 B). An inverse correlation was found between expressions of TRF2 and WHO grade (P = .008).

The Tankyrase mRNA expression was 1.28±0.58 in LGAs, 4.46±6.23 in AAs, 1.08±1.35 in GBMs. The expression levels in AAs resulted statistically higher as compared with those in LGAs (P = .009) and GBM (P<.001) (Figure 3 C). A correlation was found between expressions of Tankyrase mRNA and that of both hTERT mRNA (P = .03), and TRF-2 mRNA (P = .03).

The PARP-1 mRNA expression was 0.97±0.59 in LGAs, 17.4±5.69 in AAs, 11.5±15.5 in GBMs. The expression levels in AAs resulted statistically higher as compared with those in LGAs (P<.001). The expression in GBMs showed a tendency toward the higher expression as compared with LGAs (P = .07) (Figure 3 D).

**Figure 4:**
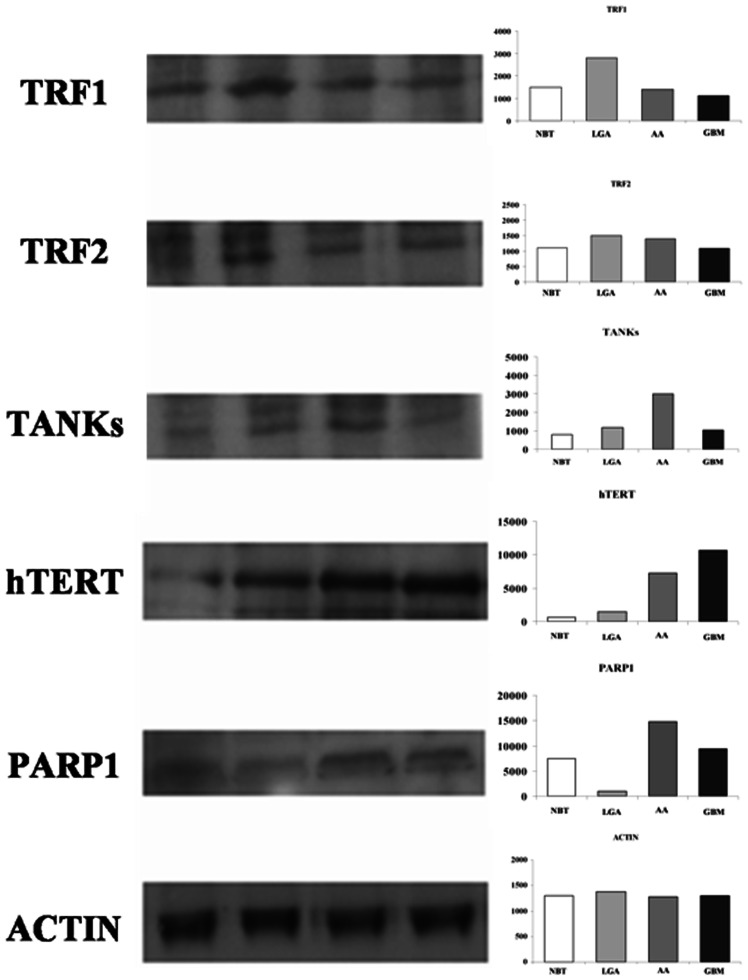
Western blot analysis of TRF1, TRF2, h-TERT, TANKs, and PARP-1. Left: representative autoradiogram; right: graphs with quantitative data. Both TRF1 and TRF2 are over expressed in LGAs as compared with AAs and GBMs. h-TERT, TANKs, and PARP1 showed a tendency toward a higher expression in malignant astrocytomas (i.e. AAs and GBMs). **Abbreviations**: NBT: Normal Brain Tissue; LGA: Low grade Astrocytoma; AA: Anaplastic Astrocytoma; GBM: Glioblastoma Multiforme; TRF1, Telomeric repeat-binding factors 1; TRF2, Telomeric repeat-binding factors 2; h-TERT human telomerase reverse transcriptase; PARP1, Poly (ADP-ribose) polymerase 1.

Western blot analysis of proteins confirmed previous results (Figure 4).

#### Possible clinical implications of telomere length, Telomerase activity and Telomere-associated proteins

A statistically significant correlation between telomere length and WHO grade was observed (P<.001). Conversely, no statistical differences between telomere length and both age and KPS, were found. A correlation was found between h-TERT and WHO grading (P<.001). No statistical differences between h-TERT and Telomere length were observed. An inverse correlation was found between expression of TRF1 and WHO grade (P = .006), and between expression of TRF2 and WHO grade (P = .008).

## Discussion

**Figure 5:**
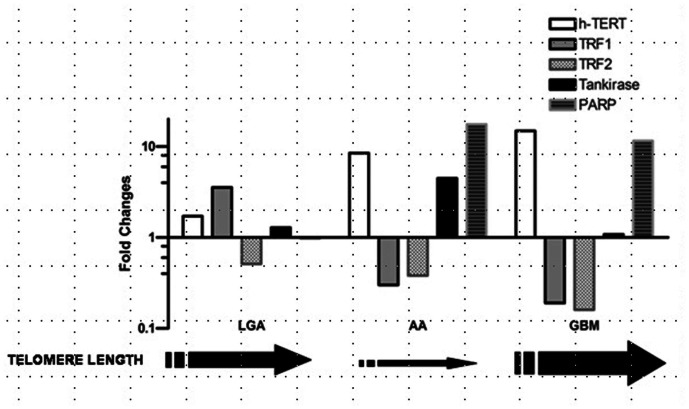
Biomolecular profiles of expression for each tumor histotype. Relative quantification of studied genes showed that expression levels of those genes are similar along with different histological grades. In LGA, an up-regulation of TRF1 occurs when telomerase has not yet been activated or is down regulated by TRF1. In AA, an up regulation of PARP-Tankirase complex and telomerase activation occurs. The ADP-ribosylation of TRFs, mediated by PARP1, diminish its ability to bind telomeric DNA, causing TRF1 down-regulation and allowing telomerase to elongate progressively telomeres. Finally, a down-regulation of TRF1 and TRF2, increasing telomerase activity, persistent over-expression of PARP-TANKs and elongated telomere are typical features in GBM. ***Black arrows*** indicates telomere length along with different stages of carcinogenesis and tumor progression in astroglial brain tumors. **Abbreviations**: LGA: Low grade Astrocytoma; AA: Anaplastic Astrocytoma; GBM: Glioblastoma Multiforme; TRF1, Telomeric repeat-binding factors 1; TRF2, Telomeric repeat-binding factors 2; h-TERT human telomerase reverse transcriptase; PARP1, Poly (ADP-ribose) polymerase 1.

Our results suggest that factors controlling telomere length are expressed at variable level in astroglial brain tumors with different grade of malignancy when compared with normal brain tissue. They also suggest the presence of a specific expression profile for different tumor grade, with telomere length depending on the balance of expression levels of different genes involved in the control of telomere maintenance. In details, up-regulation of TRF1 and 2, and shorter telomere featured LGGs, suggesting a pivotal role of these telomeric proteins in the early stage of cell immortalization. A down-regulation of TRF1 and 2, and up-regulation of both telomerase and TANK-PARP1 mainly observed in AAs and GBMs, may play a role in malignant progression of astroglial tumors toward higher malignancy levels (Figure 5).

In order to better understand the role of telomere dysfunction, including elongation or attrition, in carcinogenesis and tumor progression, we measured telomere length in astroglial brain tumor with different grade of malignancy (WHO Grade 2–4). Terminal restriction fragment measurement showed that telomere length was reduced in astroglial tumors as compared with NBT. In GBM however, telomeres were often longer than AAs and LGGs.

Telomere length is generally reduced in human tumors.[24]–[29] The relevance that this shortening plays in carcinogenesis has been extensively studied using knockout mice.[4], [30]–[32] In the absence of genome checkpoint functions, telomere dysfunction caused by telomere shortening accelerates genomic instability, facilitating cancer initiation and progression.[33]

On the other hand, the telomere length tended to increase along with malignancy of tumors.[34]–[36] Gertler et al.,[34] showed that in colorectal tumors increased TRF correlated with higher tumor stage, decreased overall survival, and in a multivariate analysis, TRF was an independent prognostic factor.

Our findings support the hypothesis that hypervariability of telomere length, already described in other human cancers, probably depends on the different stages of carcinogenesis and tumor progression. Hence, to ascertain this hypothesis, we evaluated, for the first time in gliomas, telomerase activity and the expression levels of the main genes involved in telomere maintenances including h-TERT, TRF1 and TRF2, and TANKs-PARP, in tumor tissues obtained during surgery and their paired normal tissue. In cancer cells telomere length depends on the balance between the loss of telomeric repeats during DNA replication and the elongation of telomeric repeats mediated by telomerase.[37] In most normal human somatic cells, telomerase activity is weak or undetectable. As in almost all tumors, malignant brain tumors are associated to higher telomerase activity than benign tumors, such as neurinomas, meningiomas [38] or normal brain tissue.[39] Increased telomerase expression has been also associated with higher proliferative index, tumor grading, age, vascular and endothelial proliferation,[40] poor outcome.[41]–[43] In our investigation, both telomerase activity and h-TERT mRNA levels varied in astroglial brain tumors and both were significantly correlated with tumor grading. On the other hand, no statistical correlation between h-TERT and telomere length was observed. Similar results have also been reported in other studies [37], [44] suggesting that a telomerase-independent mechanism for the regulation of telomere lengthening possibly exist in these telomerase-negative tumors.[44] Recent studies indicate that telomere-associated proteins can regulate telomerase accessibility in either positive or negative ways, suggesting a role in telomere maintenance.[45] The two major telomere- binding proteins are TRF1 and TRF2. Both may function individually or by interacting with other binding proteins, such as tankyrase, TIN2, hRap1, Mre11/Rad50/Nbs1 DNA repair complex, Ku86.[46], [47] Recent in vitro studies indicate that, overexpression of TRF1 in tetracycline-responsive human fibrosarcoma cell line HTCC75 resulted in a gradual decline in telomere length [48] and it has been reported that the forced tethering of a large number of TRF1 molecules to a single telomere induce a significant shortening of telomere length.[11] These findings suggest that in human cancer cells an upregulation of TRF1 may results in a progressive telomere shortening. Our results documented a differential telomere-associated proteins expression in astroglial brain tumors of different grade compared with normal brain tissue. An inverse correlation between TRF1 and 2, and WHO grade was also found. Several studies [48], [49] dealing with correlation of TRFs expression and telomere length in human cancers, also confirm our results. Oh et al.[49] documented that up regulation of telomere-binding proteins, TRF1, TRF2, and TIN2 is related to telomere shortening during human multistep hepatocarcinogenesis. These findings support the hypothesis that an up regulation of the telomeric binding proteins, inhibiting telomerase, results in a progressive telomere shortening and may play a role in immortalization of cancer cells. Our findings reveled a weak TA In low grade tumors, TRF1 and TRF2 mRNA is up-regulated, while TA was weak and h-TERT, tankyrase, and PARP resulted absent or minimally expressed. These data, also, confirm our previous observation that glioma cells express higher levels of TRF1 as compared with normal brain tissue. [21], [50] As expected, telomere length in such tumors was shorter as compared with normal brain tissue. In high grade tumors h-TERT, PARP and tankyrase are over-expressed with an increased telomerase activity. These findings, are consistent with the evidence of longer telomeres in those tumors.

## Conclusions

Our findings, are consistent with the hypothesis that in astroglial brain tumors, up-regulation of TRF1 and TRF2 may occur in the early stages of carcinogenesis when telomerase has not yet been activated or is down-regulated by TRF1. This stage is characterized by short telomeres, genomic instability, low proliferative rate and prolonged life span, that are typical biological behaviour of LGGs. Later, telomere dysfunction caused by telomere shortening accelerates genomic instability, facilitating cancer initiation and progression. At this stage, an up-regulation of PARP-Tankyrase complex and telomerase activation may occurs. The ADP-ribosylation of TRFs, mediated by PARP1, reduces its ability to bind to telomeric DNA, allowing telomerase to elongate progressively telomeres and extending cellular life span. Down-regulation of TRF1 and TRF2, increasing telomerase activity, persistent over-expression of PARP-TANKs and elongated telomere are typical features of GBMs. Elucidation of additional telomerase components and associated proteins will certainly contribute to further investigations of the effect of telomerase in telomeres elongation, telomere length maintenance, oncogenesis, and new unidentified cellular functions. As far as we know, no other studies regarding these issues in astroglial brain tumors are available in the literature, so further studies should be performed to better understand the pathways involved in the telomeres length maintenance and, consequently, in the process of carcinogenesis and malignant progression of human astroglial brain tumors.
